# Stochastic Heterogeneous Interaction Promotes Cooperation in Spatial Prisoner's Dilemma Game

**DOI:** 10.1371/journal.pone.0095169

**Published:** 2014-04-23

**Authors:** Ping Zhu, Guiyi Wei

**Affiliations:** School of Computer Science and Information Engineering, Zhejiang Gongshang University, Hang Zhou, Zhejiang, China; University of Maribor, Slovenia

## Abstract

Previous studies mostly investigate player's cooperative behavior as affected by game time-scale or individual diversity. In this paper, by involving both time-scale and diversity simultaneously, we explore the effect of stochastic heterogeneous interaction. In our model, the occurrence of game interaction between each pair of linked player obeys a random probability, which is further described by certain distributions. Simulations on a 4-neighbor square lattice show that the cooperation level is remarkably promoted when stochastic heterogeneous interaction is considered. The results are then explained by investigating the mean payoffs, the mean boundary payoffs and the transition probabilities between cooperators and defectors. We also show some typical snapshots and evolution time series of the system. Finally, the 8-neighbor square lattice and BA scale-free network results indicate that the stochastic heterogeneous interaction can be robust against different network topologies. Our work may sharpen the understanding of the joint effect of game time-scale and individual diversity on spatial games.

## Introduction

Cooperation is ubiquitous in nature and it plays an important role in the evolution of species [1]–[5]. However, understanding the emergence and persistence of cooperative behavior among selfish individuals remains an open and challenging problem [6], which has been widely studied by biologists, physicists and sociologists over the years [7]. Evolutionary game theory [1], [8] provides a powerful tool to investigate altruistic actions among selfish individuals and the Prisoner's Dilemma Game [9] (PDG) is a frequently studied paradigm in the field. In the original PDG, two players simultaneously choose to either cooperate (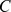) or defect (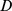). They will both get a payoff 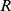 (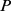) if they are both cooperators (defectors). If one cooperates and the other defects, a payoff 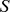 (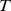) is given to the cooperator (defector). Here, the four payoff values are arranged as 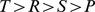 with precondition 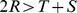. So, for a rational selfish individual, the best strategy is to defect no matter how the opponent acts. It is obvious that mutual cooperation can get a lager payoff than mutual defection, and thus there is a conflict of interest between what is best for the individual and what is best for the group. This is the so called “social dilemma”.

Traditionally, the evolutionary PDG is studied in a well-mixed population where all individuals play games with each other. It is shown that cooperation never survives evolution and defectors can diffuse in well-mixed populations under replicator dynamics [5]. However, observations in the real world usually show the opposite. To explain the emergence and maintenance of cooperative behavior, several mechanisms have been proposed, such as kin selection [10], direct [1] or indirect [11] reciprocity, punishment [12], reputation [13], group selection [14], voluntary participation [15], payoff aspiration [16] and so on (see Ref. [7] and *infra*).

Since Nowak and May [17] introduced spatial structure into the evolutionary game, it has increasingly attracted interest from different fields (see Ref. [18], [19] and *infra*) as a significant extension of the traditional well-mixed territory. In spatial games, the nodes in the network indicate the game players and the edges linking two players mean the interaction between them. Fruitful studies have shown that the network topology [20], [21] provides a powerful boost in the evolution of cooperation, e.g., regular networks [22]–[29], small-world networks [30]–[35] and scale-free networks [36]–[42]. Recently, the player mobility [43]–[47], the adaptive networks [48], [49], as well as co-evolutionary strategy and network structure [50], [51] also exhibit promising influence on cooperative behavior.

As we know, both game time-scale [52] and individual diversity [53] play important roles in the promotion of cooperation. In terms of time-scale, Carlos theoretically studied the effect of time-scales of interaction and selection on the outcome of evolution [52]. Szolnoki [54] studied the effect of time separation by introducing a simply co-evolutionary rule where the teaching activity of a successfully reproduced player is increased. Wu [55] studied the diversity of reproduction in time-scale models and found that the cooperation level in spatial PDG is greatly promoted. Tanimoto [56] studied the effect of strategy updating time scale on network reciprocity. They found that a negative correlation between degree and strategy updating speed brings an extremely large cooperation-enhancing effect. Rong [57] studied an adaptive strategy selection time-scale called “win-slower, lose-faster” rule. Traulsen [58] studied the heterogeneity of game time-scale. Chen's work [59] found that interaction stochasticity supports cooperation in spatial PDG. Jiang studied the effect of time-scale in mobile environments [60]. In particular, Cao [61] studied two time-scales in a dynamic weighted network, i.e., the strategy update time-scale and weight adjustment time-scale. On the other hand, since the prestigious work in [62], individual diversity is becoming one of the hottest focuses. Santos [53] pointed out that diversity can be the diverse nature of human interaction, contexts, preferences and social structures. Szolnoki [63] studied the inhomogeneous teaching activity controlled by a two-value pre-factor which is determined according to the type of the player whom the strategy learned from. In [64], the authors further studied the effect of teaching activity which is individual diverse and time evolving. Yang [65] analyzed individual heterogeneity in scale-free structures. Wu [66] investigated the evolutionary PDG with dynamic preferential selection and found that the proposed mechanism substantially promotes cooperation. Perc [67] studied the effect of social diversity on cooperative behaviors in PDGs, where different distributions are introduced to determine the social diversity of players. Wang [68] found that heterogeneous aspirations can promote cooperation in the game. Zhang found that cooperation level can be remarkably promoted when the heterogeneity of view radii is considered [43]. Cao [42] concerned investment heterogeneity in scale-free structured networks and proposed a PGG model to study the effect of investment heterogeneity on cooperation level. Zhang [69] investigated the evolution of cooperation on scale-free networks with heterogeneous payoff allocation mechanism. Shigaki [70] studied another interesting form of diversity, i.e., the initial fraction of cooperators. As their work shown, different initial proportion of population results in different END and EXP behaviors as well as distinctive evolutionary snapshots and final equilibrium. Tanimoto [71] studied the heterogeneous aspiration in partner selection process and found that cooperation level is remarkably enhanced by spatially distributed updating speed among agents. A recent work by Sirakoulis [72] also studied heterogeneity of the enhancement factor 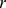 in PGG in a power-aware embedded system. To name but a few, the heterogeneity of population acts as an important role on players cooperative behavior (see Ref. [53] and *infra*).

However, to the best of our knowledge, joint research of time-scale and individual diversity has not been studied before. As we know, in the real world, not all interactions are effective [59] and individuals always act heterogeneously [53]. Thus, as a natural extension of previous works, in this paper, we explore the effect of stochastic heterogeneous interaction on players' cooperative behavior. As for “stochastic”, we mean that the interaction between each pair of players is randomly occurring; in terms of “heterogeneous”, we suggest that the randomly-occurring interactions may accord with certain distributions globally. A detailed discussion on interaction modes can refer to Refs. [73]–[75] and *infra*.

This paper is organized as follows: in section 2, we describe the model used in this work; section 3 presents the simulation results and analysis; in section 4, we compare our result with some previous works and finally conclude the paper.

## Methods

In our work, we adopt the evolutionary PDG on a 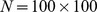 4-neighbor square lattice with periodic boundary conditions. Here we consider the re-scaled payoff matrix:
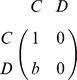
Namely, 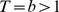, 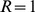, 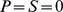, where 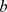 is the temptation to defect.

Initially, all players choose their strategies at random, i.e., cooperation (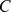) or defection (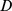). Here, we use 0 to represent cooperation and 1 for defection. Then, each player interacts with its four nearest neighbors on the topology structure. Note that not all the interactions are always in effect, a probability 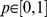 is introduced to conduct the game time-scale in each generation of PDG interaction. Moreover, the diversity of time-scale is described by following distributions [43], [67]:
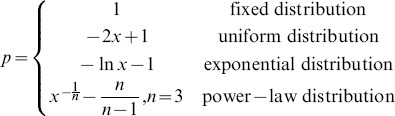
(1)where 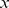 are random numbers following a uniform distribution with unit interval. The resulting value of 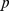 generated from Eq.(1) is not guaranteed to locate within [0,1], and is solved by taking some normalization methods. Thus the “stochastic heterogeneous interaction” can be stated as each interaction occurring with random probability, and all these 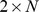 probabilities obeying certain distributions (e.g., fixed, uniform, exponential and power-law distribution) in the region of [0,1].

After playing one shot of the game, each player will learn from one of its adjacent neighbors and update its strategy. As reported in Ref. [76], players have different attractiveness in society. So when players choose neighbor for updating strategy, we consider individual's inhomogeneous attractiveness which is in proportion to their payoffs. We define the selection probability of player 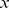 selecting one of its neighbors, 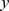 as following function:
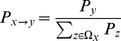
(2)where 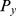 represents the payoff of player 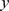, and 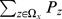 means the sum of payoffs running through all the neighbors of 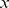. If 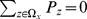, we solve the problem with a solution in which player 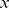 randomly chooses one player 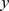 from its neighbors. The partner selection process is unequivocally important to the promotion of cooperation. In realistic scenario, for example, people are much more likely to follow a successful individual than someone who is struggling to get by. This implies that more powerful neighbor (e.g., with larger payoff) gets a bigger chance to be chosen. While under certain circumstances, it is also possible that individuals will be inspired to copy their less successful partners. Thus, a tunable factor should be defined to control these situations [68], [71], [77]. In our work, we focus the highlight on intermittent gaming (e.g., stochastic heterogeneous interaction), thus adopting the relative simple partner selection rule depicted in [76], i.e., the probability of selection is positive correlation with payoff.

Next, player 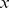 adopts the selected neighbor's strategy with a probability calculated by the following function [78]:
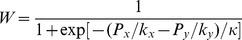
(3)Here, 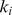, 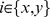, indicates the degree of player 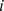, 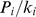 means the average payoff of player 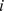 and 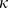 indicates the noise effect. Following [78], we set 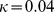.

## Results and Analysis

In what follows, we show the simulation results carried out on a 4-neighbor square lattice of size 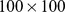. At the beginning, the strategies of cooperation and defection are randomly distributed in the population with an equal probability of 0.5. The key level of cooperation is characterized by the fraction of cooperators 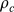 in the whole population. In the following simulations, 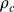 is obtained by averaging over the last 1,000 generations of the entire 10,000 generations. 300 individual runs are subsequently averaged together to eliminate stochasticity and return the final datum.

**Figure 1:**
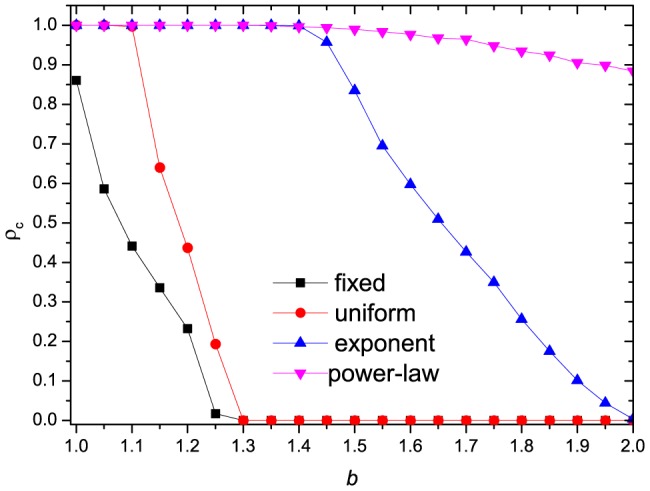
The cooperation level as a function of 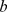 for different distributions of 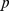, i.e., fixed, uniform, exponential and power-law distribution.

First we present the cooperation level as a function of temptation 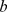 for different distributions of 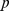, as shown in Figure 1. We find that, as 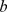 increases, cooperation level will decrease or vanish irrespective of different interaction distributions. For power-law distribution, the cooperation always stays at a high level, even when 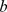 approaches 2.0, about 90% of cooperators still survive. Although cooperation level decreases in the region of 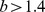, the slope is smooth. For exponential distribution, cooperation drops gradually when 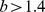. We can also find that, for uniform distribution, the cooperation levels decline to zero sharply, and when 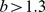 the system is filled with defectors. In case of fixed distribution, i.e., without game time-scale and 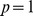, even when 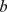 is very small, there are still some defectors in the system; while in the other three distributions, the system is in all-cooperators state when 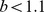. The rank of the effect of promoting cooperation of four discussed distributions is arranged as 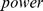-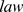 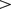 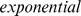 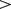 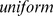 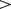 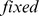.

**Figure 2:**
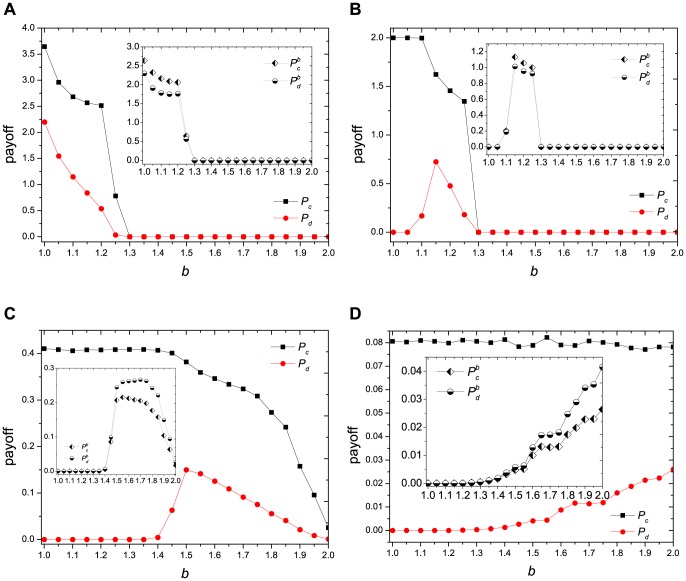
The mean payoffs of cooperators and defectors in the whole population and along the boundary, respectively obtained by simulations as a function of 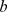, for four different interaction distributions. (a) fixed distribution, (b) uniform distribution, (c) exponential distribution and (d) power-law distribution.

To qualitatively explain the nontrivial results in Figure 1, we first study the relationship of 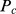, 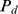, 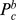,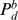 in the equilibrium state, where 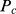 (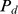) stands for the mean payoffs of cooperators (defectors) in the whole population; 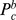 (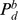) represents the mean payoffs of cooperators (defectors) along the cooperator-cluster (defector-cluster) boundary. The results are summarized in Figure 2. For all the four graphs describing the mean payoffs, the cooperators obtain a larger payoff than defectors. The four inset graphs describe the mean boundary payoffs. In fixed distribution (Figure 2(a)) and uniform distribution (Figure 2(b)), the mean boundary payoffs of cooperators are larger than those of defectors; while in exponential distribution (Figure 2(c)) and power-law distribution (Figure 2(d)), 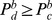 holds. As Figure 2 shows, in fixed distribution, as 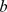 increases, 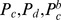 and 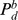 monotonously decline to zero. And when 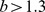, the system is full of defectors, resulting in 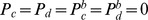. In uniform distribution, when 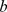 is small, the system is saturated with cooperators, and 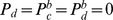. As 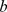 increases, the number of defectors (cooperators) increases (decreases), and when 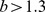, the system is full of defectors, which is similar to the fixed distribution. Notice that in the region of 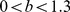, 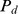 increases to a local maximum and then decreases to zero, this is caused by evolution of mean cooperator neighbors of defectors. In exponential distribution, when 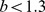, the system is filled with cooperators, thus 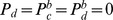. As 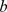 increases, the defectors take over neighboring cooperators gradually and finally seize the whole network. Here, there is also a local maximum of 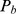 when 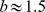. In power-law distribution, the cooperators and defectors coexist when 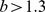.

**Figure 3:**
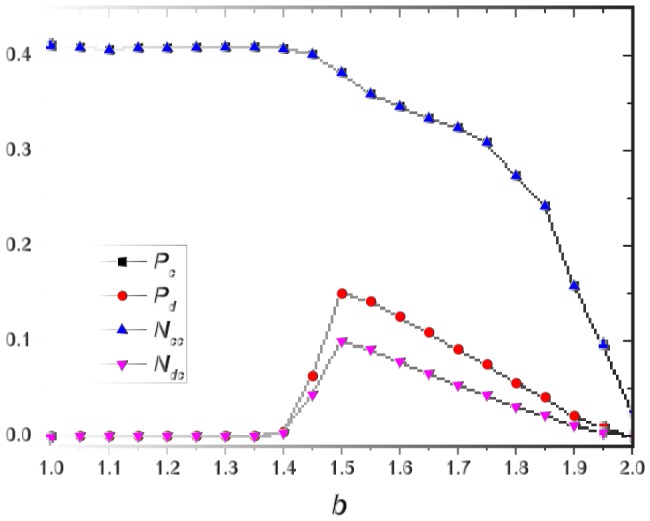
The mean number of cooperator's (defector's) cooperative neighbors as a function of 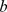 underlying exponential distribution.

**Figure 4:**
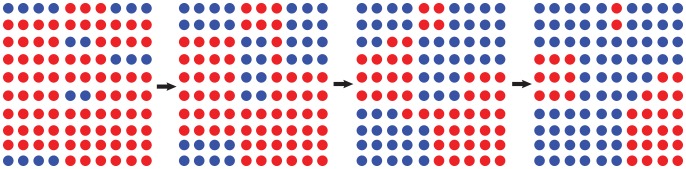
The evolution of 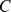-cluster (red) and 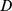-cluster (blue) in a 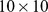 square lattice.

According to Figure 2, the cooperative behavior in Figure 1 can be explained as follows (taking Figure 2(c) for example): the mean payoff of cooperator (defector) 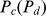 can be calculated as:
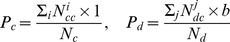
(4)where 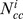 (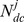) is the total number of cooperator 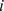
's (defector 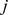
's) cooperative neighbors and 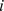 (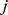) goes through all 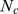 cooperators (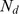 defectors). Rewriting Eq.(4), we get
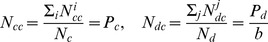
(5)where 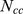 (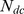) is the mean number of cooperator's (defector's) cooperative neighbors. The mean number of cooperator's (defector's) cooperative neighbors as a function of 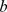 underlying exponential distribution is shown in Figure 3. From Figure 3, we can see that as 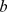 increases, the mean number of cooperator's cooperative neighbors 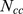 decreases. As the 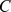-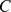 link occurs only inside of 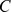-clusters, the decrease of 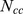 indicates that a 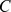-cluster is shrinking. On the other hand, because the 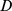-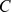 link occurs only along of the boundaries of 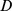-clusters, so the increase of 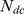 in the region of 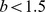 indicates the expansion of 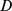-cluster. However, after a local maximum of 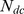, the mean number of defector's cooperative neighbors gradually decreases to zero. We contend that after the local maximum of 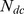, the 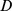-cluster is still expanding, however the behavior is different. The adjacent expanding 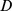-clusters merge into a larger 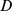-cluster, and thus 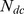 decreases. The evolution of 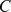-cluster and 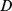-cluster is described in Figure 4.

**Figure 5:**
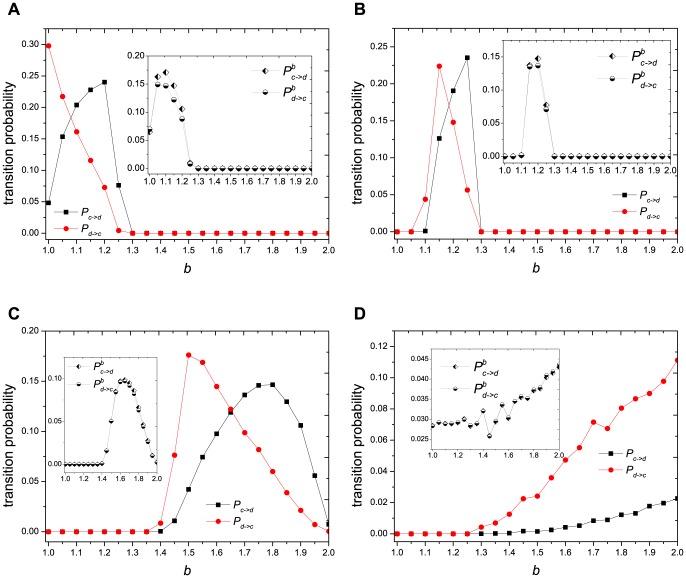
The probability of cooperators and defectors transmuting into each other, as a function of 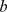. (a) fixed distribution, (b) uniform distribution, (c) exponential distribution and (d) power-law distribution. The inset graphs are the (b) corresponding boundary transition probabilities.

To carry further our description of the evolutionary behavior, we draw the transition probabilities between cooperators and defectors by increasing 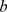 for different interaction distributions in Figure 5. Here we use 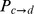 denoting the transition probability from cooperators to defectors and 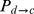 denoting the opposite transition probability. The inset graphs in each sub-figure correspond to the boundary transition probability of cooperators to defectors (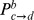) and defectors to cooperators (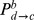). The dynamic evolution process can then be described as follows (taking Figure 5(c) for example): firstly, the system is full of cooperators, thus 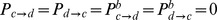. As 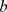 increases, transitions between cooperators and defectors coexist. However, we now have 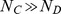, and thus leads to 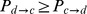. At this time, small portion of defectors are almostly surrounded by cooperators, and thus the defectors' payoffs increase (see Figure 2(c)), which results in 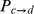 increasing simultaneously. The local maximums of 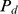 in Figure 2(c) and 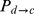 in Figure 5(c) indicate the turning point where the defectors, who are spreading over the ocean of cooperators, now have the maximum number of mean cooperative neighbors, and after which both of them will decrease. However, the curve of 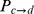 will keep on increasing after the local maximums of 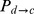. This is because the local maximum of 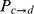 will locate at the maximum value of 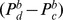 (see the inset graph of Figure 2(c)). The crossing point of 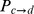 curve and 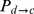 curve indicates the state of 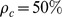, after which defectors will outperform cooperators, and thus 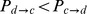.

To sum up, the answer to why the rank *power-law* 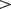 *exponential* 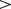 *uniform* 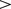 *fixed* holds is stated as follows: (**I**) *fixed* 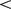 *uniform*. We contribute the cooperation rate improvement to the introduction of our stochastic heterogeneous interaction mechanism. In particular, by introducing game time-scale, the mean probability of game interaction becomes 50% in uniform distribution. So, compared with the fixed case, the payoffs for cooperators and defectors are also reduced to 50%. Thus, according to the update rule, i.e., Eq.(3), the transition probability 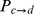 (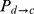) decreases (increases), resulting in an improvement of 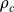. Similarly, we can also prove *fixed* 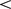 *exponential* and *fixed* 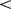 *power-law*. (**II**) *uniform* 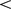 *exponential*. In exponential distribution, it is shown that the decreasing slope to the all-defectors state is smoother and slower than in uniform distributions. This is because defectors lying around boundary in exponential distributions hold a payoff advantage over cooperators (see inset graph of Figure 2(c)). As we know a defector will have a higher payoff if he is surrounded with cooperators. In exponential distributions, we have 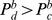, this implies that a certain number of boundary cooperators exist, which slows down the decrease of cooperation rate. (**III**) *exponential* 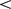 *power-law*. We argue that the joint effect of 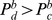 and 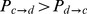 results in a high final cooperation level. As in exponential distribution, 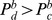 slows down the transition from cooperators to defectors; and 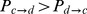 makes cooperators keep their advantage over defectors (i.e., according to Figure 5, before the 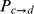 curve and 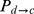 curve cross, we have 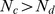 and 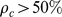).

**Figure 6:**
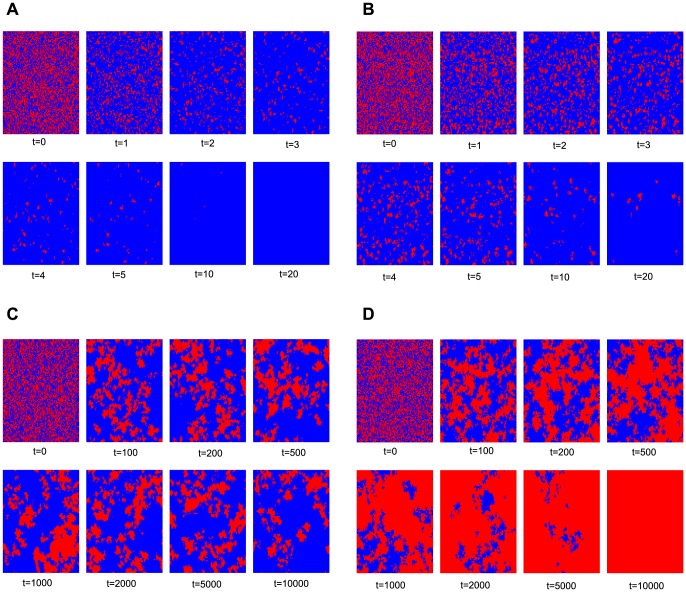
Snapshots of typical distributions of cooperators (red) and defectors (blue) on 4-neighbor square lattice for 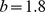 and four different interaction distributions. (a) fixed distribution, (b) uniform distribution, (c) exponential distribution and (d) power-law distribution.

**Figure 7:**
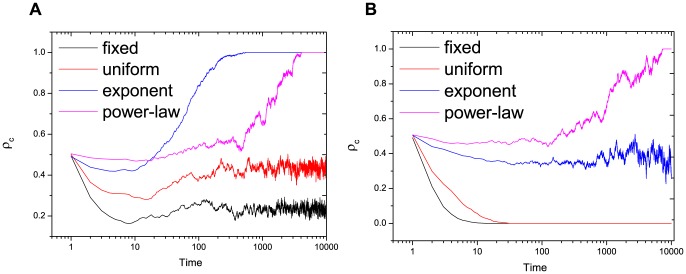
Evolution time series for 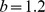 (a) and 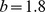 (b) of fixed, uniform, exponential and power-law distributions.

We then investigate the cluster formation process at different time step 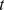 for 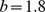 and four different interaction distributions, as plotted in Figure 6. At the beginning, cooperators and defectors are randomly distributed on the square lattice with equal probability. For fixed distribution, as 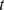 increases, the size of a cooperator cluster decreases quickly and vanishes at discrete time step 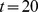. For uniform distribution, the evolution process is slower than fixed distribution, i.e., fewer cooperators are distributed through the population when 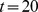. For exponential and power-law distributions, the sizes of cooperator clusters are larger. The power-law distribution has greater effect on the formation of cooperator clusters than exponential distribution and its cooperators occupy the whole population when time step 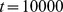. Figure 7 shows the evolution time series for 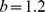 and 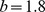 of the four distributions, respectively.

**Figure 8:**
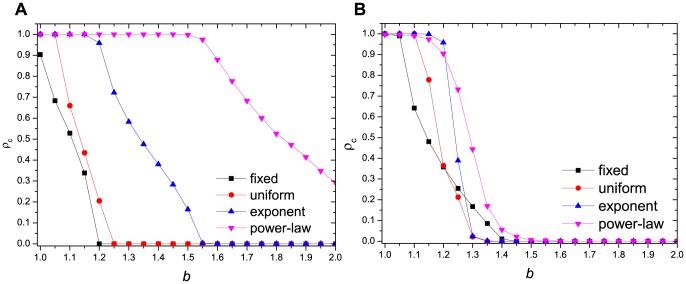
Simulation results of our stochastic heterogeneous interaction on: (a) 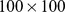 regular lattice with Moore neighborhood; (b) Barabási-Albert scale-free network with network size 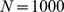 and 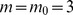.

To explore the robustness of our stochastic heterogeneous interaction over different network topologies, we examine the cooperative behavior on a regular lattice with Moore neighborhood (i.e., average connectivity 8) [e.g., Figure 8(a)] and on a Barabási-Albert scale-free network [62] [e.g., Figure 8(b)] with different values of 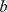. We find that the general cooperative tendency is the same as that shown in Figure 1, indicating that our stochastic heterogeneous interaction can be robust against different network topologies.

## Discussion

In general, players achieve payoffs through interaction with their neighbors, either locally or globally. More precisely, in our work, an interaction process can be further divided into game-step (which generates payoffs) and reproduction-step (which generates offspring). The speed (time-scale) of game and reproduction is always assumed to be equal. However, in natural systems, individuals do not always deterministically interact with others in the population, e.g., an individual can interact many times before the end of its lifetime [55]. In this situation, the speed of reproduction is slower than that of game. In [54], the co-evolutionary parameter also acts as the role of reproduction time-scale. In [56], distributed reproduction time-scale is studied. In [57], a coevolving reproduction time-scale is studied. On the other hand, the speed of reproduction can also be faster than game. Previous works [58], [59] introduce game-step time-scale to achieve payoff heterogeneity, however the realistic significance of such assumption has not been indicated. In [60], the authors suggest a reasonable assumption for game-step time-scale: when it's time to play game with neighbors, the player has multiple choice, i.e., with probability 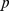 to play game and with probability 1-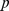 to migrate. In [74], [75], the reputation-based conditional interaction is also another form of game-step time-scale. No matter what external forms they present, theses previous works [54]–[60] have one thing intrinsically in common: the time-scale is node-level applied. The probability, either in game step or reproduction step, reflects the willingness of the player. In reproduction step, this willingness is unidirectional. Reproduction will happen as long as the probability is satisfied. However, in game step, this willingness must be bidirectional, i.e., the game will not take place until both of the player accept the probability (game-step time-scale) of playing the game [43], [74], [75]. Our model extends the node-level time-scale to the edge-level time-scale in game-step aspect. In our work, not node, but each edge is assigned with a probability. This simple setting makes our model distinctive and powerful. The game proceeds under the guidance of the probability attached with each edge. The player can be relaxed from calculating the tolerance (or range) of the game. Actually, it is hard to formulate convictive criteria to decide whether to play the game in node-level time-scale. To help understand the intrinsic meaning of edge-level time-scale, one can draw such a scenario: an 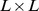 lattice made of conductor, and it is DC charging. Moreover, each edge can be replaced with a nonconductor. As a result, when the edge is made of conductor, there is current going through; when the edge is replace with nonconductor, the current is blocking. The probability of what material the edge is made of indicates the edge-level time-scale. In the real world, the semiconductor can also be considered as a special kind of edge-level time-scale.

In our model, “stochastic” means that the interaction between each pair of players is randomly occurring. Each edge in the system is attached with a random probability. This setting also distinguishes our work from the previous ones. In works [55], [58]–[60], the time-scale is fixed for all the nodes in the system during the evolutionary process. In works [54], [57], [74], [75], the time-scale is initially set as constant. However, the authors introduced co-evolutionary strategies to make the time-scale stochastic. In our work, the time-scale is initial randomly distributed, and as the game proceeds, they do not change. But we carry 300 individual runs (i.e., 300 different sets of time-scale probability) and average together to get the final datum. From this perspective, we can say the time-scale changes in our work as well. It should point out that our work differs from Tanimoto's [56] in the aspect that we focus on the game-step time-scale while their work concerns the reproduction time-scale. Further work may introduce co-evolutionary strategy on edge-level time-scale into our stochastic heterogeneous interaction model. Note that in some work, although a probability parameter is discussed, it is not within the category of time-scale [73]. As we know, the interaction patterns is an important element behind the emergence of cooperation [66]. Traditionally, there are two types of networks determining the population structure: the interaction network and the replacement network. The interaction network focus on the game step, i.e., with whom individual will play game; while the replacement network cares about from whom one will learn strategy in the update process. In [73], the probability control the game or reproduction range of the player, the speeds of game and reproduction are set as equal and the total number of opponents is invariable. As far as time-scale concerns, we believe it is meaningless to study game-step time-scale and reproduction time-scale jointly. Indeed, a ratio between game and reproduction time scales is enough [52]. When this ratio is greater than 1, it corresponds to the game time-scale being faster than the reproduction time-scale, and vice versa. When this ratio approaches to zero, the game recovers the round-robin procedure. It should point out that the two time-scales studied in [61] is different from previous works as well as our work. In their paper, 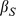 is reproduction time-scale. However, the other time-scale 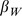 is not game-step time-scale; it is the time-scale for network adaption. So their joint research of both the two parameters is not redundant. It's also worth mentioning that the reproduction time-scale can only be node-level, or the node-level is enough. As we pointed, unlike game time-scale, which must get approval from both sides, the reproduction process is individual's unilateral willingness.

As we know, cooperation among individuals can be enhanced by diversity among them. There always exists a hub node to attract and maintain cooperative clusters. One form of diversity is represented by well-designed functions [42], [63]–[66], [68], [69]. In this area, researchers mainly concentrate on the relationship between the degree of diversity and the population's cooperation degree. It is found that too much diversity will not promote cooperation, because the defectors will take over the hubs, and there exists an optimal level of diversity for maximal degree of cooperation. Different from these works, our “heterogeneous” is characterized by stochastic distributions motivated by some previous works. In [43], [67], the authors add stochastic vibrations to the basal value. The vibration can be both negative and positive, and the integral average of vibration is restrained to be zero. Similarly we stochastically generate the probability set. However, in our model, the generated heterogeneity must be positive. Moreover, in addition to guarantee the integral average of probability be zero, their value must lie in the closed interval of [0,1]. Interestingly, we observed that the rank of the effect of promoting cooperation is arranged as *power-law* 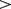 *exponential* 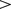 *uniform* 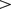 *fixed*. It is conceptually analogous to the observations in [43]. Our result is also in accordance with scale-free diversity studies [36]–[42], which is explain through Figures 2–6 in detail. It's also worth pointing out that different from previous works, in our model the heterogeneity is edge-level applied. We say that our interaction mode is also edge-level executed. In node-level game models, during the interaction process, we pick up individuals, and let them play game with their neighbors. The total number of interaction times is calculated as 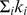. Here 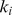 is the degree for individual 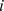, and 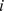 runs through all the nodes in the system. In edge-level game model, we pick up edges, and let the two individual attached play the game. The total number of interaction times is 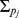. Here 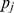 is the edge-level time-scale, i.e., Eq. (1), and 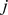 runs through all the edges in the system.

It is worth mentioning that the PD game we discussed in the paper is the so-called weak prisoner's dilemma. Under this model, we control the dilemma strength by a single parameter *b*, which simplifies our discussion. But, we have to admit that this particular game class is limited, since there is only Chicken-type dilemma but none of Stag Hunt-type dilemma [79], which might cause our results be less universal. Further work could reference to Donor Recipient (D&R) games, where 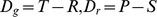 and 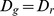 [56], [70], [71] is assumed.

One more thing to clarify is the reciprocity between intermittent gaming (e.g., our stochastic heterogeneous interaction) and partner selection rule (e.g., Eq.(2)) in the paper. As we know cooperative individuals can form 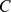-clusters to prevent the inner from being invaded by outside defectors. Here, along the boundary of 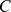-clusters, a “moat”, i.e., the edge-level time-scale, is guarding. Such moat can slow down cooperators' interaction frequency with the outside detectors; sometimes even isolate their communication, e.g., 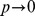. On the other hand, the partner selection rule determines individual's opponent from whom the strategy is learned. In our model, the probability of selection is positive correlation with payoff. That is to say, if a defective neighbor has higher payoff than a cooperative neighbor, then the former will be chosen for updating. When 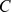-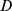 game happens, the defector gains larger payoff than cooperator, e.g., 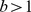. However, controlled by our stochastic heterogeneous interaction, the frequency of 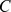-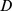 game is reduced, which consequently decreases defector's accumulated payoff. Thus, the probability of selecting a defective neighbor is reduced, the 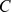-cluster survives and holds under the jointly effect of stochastic heterogeneous interaction and partner selection. The mechanism can be summarized as follows: “stochastic heterogeneous interaction” isolates individuals 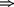 defectors' payoff is reduced 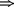 “positive correlated partner selection rule” prefers to choose cooperators 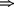 cooperation survives and maintains.

In summary, we have studied the effect of stochastic heterogeneous interaction on the evolution of cooperation with spatial prisoners dilemma game model. We assume that the interaction between each pair of players happens with a probability, and the probability satisfies certain distribution. The simulation results show that heterogeneous interaction probability can promote cooperative behavior. The rank of the effect of promoting cooperation of four discussed distributions is arranged as 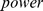-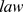 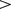 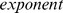 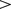 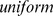 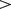 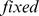. We also find that the stochastic heterogeneous interaction can be robust against different network topologies. The most significant contribution of the paper is the discussion of edge-level time-scale. In future work, it would be interesting to introduce co-evolutionary strategy on time-scale into our stochastic heterogeneous interaction model.
